# Association Between Thyroid Hormones, Thyroid Antibodies, and Cardiometabolic Factors in Non-Obese Individuals With Normal Thyroid Function

**DOI:** 10.3389/fendo.2018.00130

**Published:** 2018-04-05

**Authors:** Jia Liu, Yan Duan, Jing Fu, Guang Wang

**Affiliations:** Department of Endocrinology, Beijing Chao-yang Hospital, Capital Medical University, Beijing, China

**Keywords:** autoimmune thyroiditis, cardiovascular disease, atherosclerosis, chronic inflammation, insulin resistance

## Abstract

**Background:**

Hypothyroidism is an important risk factor for cardiovascular diseases, and autoimmune thyroiditis (AIT) is the leading cause of hypothyroidism. Recent studies showed that even AIT patients with euthyroidism still had an increased number of early atherosclerotic lesions. However, the precise mechanism is not yet known. This study aimed to investigate the association of thyroid function, thyroid autoimmunity, and cardiometabolic risk factors in non-obese AIT patients with euthyroidism.

**Methods:**

A total of 5,608 non-obese individuals including 1,402 AIT patient and 4,206 sex-, age-, and body mass index (BMI)-matched healthy controls were recruited.

**Results:**

The AIT patients had significantly lower free T3 and free T4 levels, and higher TSH, antithyroid peroxidase antibodies (TPOAb) and TgAb levels. The elevated levels of high sensitivity C reactive protein (hsCRP) and homeostasis model assessment of insulin resistance (HOMA-IR) were observed in the AIT patients than the controls [hsCRP: 0.65 (0.27–1.33) vs. 0.20 (0.03–0.74) mg/L; HOMA-IR: 2.78 ± 1.60 vs. 2.33 ± 1.49; all *P* < 0.05]. Thyroid function was not associated with metabolic parameters and inflammatory makers, while the TPOAb titer was positively associated with the HOMA-IR and hsCRP levels after adjustment for confounding factors (all *P* < 0.05). Multivariate regression analysis demonstrated that the TPOAb level was an independent influencing factor for the HOMA-IR and hsCRP levels (HOMA-IR: β = 0.058, *P* < 0.05; hsCRP: β = 0.108, *P* < 0.05).

**Conclusion:**

The TPOAb level is associated with HOMA-IR and hsCRP levels independently of thyroid function in non-obese individuals. Mild deviation of thyroid function within the normal range, chronic inflammation, and insulin resistance may be the links between AIT and atherosclerosis in the non-obese population.

## Introduction

Autoimmune thyroiditis (AIT) is a common endocrine disease characterized by diffuse thyroid enlargement and lymphocytic infiltration (1, 2). AIT manifests in patients through different states of thyroid function, such as euthyroidism or subclinical or overt hypothyroidism (1). Subclinical or overt hypothyroidism is an important risk factor for cardiovascular diseases (3). Many studies, including our previous studies, found that hypothyroidism causes insulin resistance, dyslipidemia, and chronic inflammation and further contributes to the development of atherosclerosis (4, 5). Notably, evidence from recent studies has shown that even AIT patients in euthyroid still have increased number of early atherosclerotic lesions (6, 7). However, the precise mechanism has not yet been revealed. Previous studies have shown that euthyroid AIT patients have relatively higher TSH levels than controls, and the TSH levels were positively associated with insulin resistance (8–10). Moreover, elevated total cholesterol (TC) levels were also present in euthyroid AIT patients (11). However, these results are inconsistent, and most of this evidence comes from an obese population. Obesity is a primary risk factor for insulin resistance and a chronic inflammatory state, and also affects thyroid function (12–15). Therefore, obesity might influence the reliability of results. This study aimed to investigate the association of thyroid function, thyroid autoimmunity, and cardiometabolic risk factors in non-obese AIT patients with euthyroidism.

## Materials and Methods

### Study Population

A total of 1,402 non-obese AIT patients were recruited from Endocrinology Department of Beijing Chao-Yang Hospital affiliated with Capital Medical University from June 2015 through July 2016. Every AIT patient was matched by three sex-, age-, and body mass index (BMI)-matched healthy individuals who were recruited from the Physical Examination Center of Beijing Chao-Yang Hospital Affiliated to Capital Medical University. Finally, 4,206 sex-, age-, and BMI-matched healthy individuals were included as the control group. Non-obese individuals were diagnosed if their BMI < 30 kg/m^2^. Oral glucose tolerance tests, thyroid function tests, and thyroid ultrasonography were performed at screening. Individuals with thyroid dysfunction, hypertension, diabetes, coronary artery disease, liver or renal function impairment, infectious disease, systemic inflammatory disease, or cancer were excluded. No subject took lipid-lowering agents, levothyroxine, or antithyroid drugs. Participants who were pregnant, possibly pregnant, or ingesting agents known to influence thyroid function were also excluded. Subjects with an abnormal free T4 (FT4) concentration (reference range: 12.00–22.00 pmol/L) or TSH concentration (reference range: 0.51–4.94 IU/mL) were excluded. AIT was diagnosed by elevated antithyroid peroxidase antibodies (TPOAb) (reference range: 0.00–60.00 U/mL) and/or antithyroglobulin antibodies (TgAb) (reference range: 0.00–60.00 U/mL) and typical hypoechogenicity of the thyroid in a thyroid ultrasound (6). All enrolled subjects provided written informed consent, and the protocol of this study was approved by the Ethics Committee of the Beijing Chao-Yang Hospital, Capital Medical University.

### Clinical and Biochemical Measurements

A standard questionnaire was used to collect information about each patient’s health status and medications. Height and weight were measured to the nearest 0.1 cm and 0.1 kg by the same trained group, respectively. Venous blood samples were obtained after overnight fasting. Blood samples of all participants were stored at −80°C. High-density lipoprotein cholesterol (HDL-C), low-density lipoprotein cholesterol (LDL-C), triglyceride (TG), and TC levels were measured by colorimetric enzymatic assays using an autoanalyzer (Hitachi 7170). Serum TC was measured by an enzymatic cholesterol oxidase reaction, TG by a glycerol lipase oxidase reaction, and HDL-C and LDL-C were measured by a direct assay. Fasting blood glucose (FBG) was measured by the glucose oxidase method, and fasting insulin (FINS) by the chemiluminescence method. High sensitivity C reactive protein (hsCRP) was measured with an immunonephelometric assay. Free T3 (FT3), FT4, and TSH were determined by electrochemiluminescence immunoassay using an Abbott Architect i2000 (Abbott Diagnostics, Abbott Park, IL, USA), and the intra- and interassay coefficient of variation was below 10% for FT3, FT4, and TSH. The serum concentrations of TgAb and TPOAb were detected by chemiluminescent immunoassay. For both TgAb and TPOAb, the intra- and interassay coefficient of variation was below 10%. BMI was calculated as the weight in kilograms divided by the height in square meters. Homeostasis model assessment of insulin resistance (HOMA-IR) was performed to evaluate insulin resistance according to the following formula: HOMA-IR = [FINS (μIU/mL)*FBG (mmol/L)/22.5] (16). Thyroid ultrasound was assessed by a well-trained ultrasound physician.

### Statistical Methods

Normally distributed variables were expressed as mean ± SD, while variables with a skewed distribution, including TG, FINS, TSH, TPOAb, and TgAb, were given as median and upper and lower quartiles. Variables that were not normally distributed were log-transformed before analysis. The differences between two groups were analyzed by independent Student’s *t*-test or the Mann–Whitney *U* test. The proportions were analyzed using chi-square tests. The differences between groups were analyzed by ANOVA test. We also performed Pearson and Spearman correlation analyses. Bonferroni correction was further made for multiple correlations. Multivariate stepwise regression analysis was used to evaluate the relationship between parameters. All statistical analyses were performed with SPSS 17.0 (SPSS, Inc., Chicago, IL, USA), and the results were considered statistically significant with two-tailed analyses, *P* < 0.05.

## Results

### Clinical Characteristics of the Control and AIT Groups

The clinical characteristics of the participants are summarized in Table 1. The control and AIT groups did not significantly differ in age, gender, BMI, TC, LDL-C, HDL-C, and TG. The levels of FBG and FINS were also statistically the same between the two groups. AIT patients had significantly lower FT3 and FT4 levels, and higher TSH, TPOAb, and TgAb levels, compared with the controls [FT3: 4.94 ± 0.58 vs. 5.15 ± 0.58 pmol/L; FT4: 15.76 ± 2.00 vs. 16.17 ± 1.92 pmol/L; TSH: 2.11 (1.47–2.98) vs. 1.62 (1.20–2.20) mIU/mL; TPOAb: 135.75 (46.70– 1,248.70) vs. 29.10 (0–38.00) IU/mL; TgAb: 131.90 (61.30–241.00) vs. 15.60 (0–22.80) IU/mL; all *P* < 0.01; Table 1]. The increased levels of hsCRP and HOMA-IR were observed in the AIT patients compared with the controls [hsCRP: 0.65 (0.27–1.33) vs. 0.20 (0.03–0.74) mg/L; HOMA-IR: 2.78 ± 1.60 vs. 2.33 ± 1.49; all *P* < 0.05; Table 1].

**Table 1:** Clinical characteristics of the control and AIT groups.

Parameters	Control group (*n* = 4,206)	AIT group (*n* = 1,402)	*P*
Age, years	45.50 ± 12.56	45.97 ± 12.08	0.128
Gender, F/M	3,098/1,108	1,036/366	0.334
BMI, kg/m^2^	23.98 ± 2.70	24.01 ± 3.18	0.740
TC, mmol/L	5.01 ± 0.92	5.02 ± 0.97	0.739
LDL-C, mmol/L	2.73 ± 0.73	2.72 ± 0.74	0.538
HDL-C, mmol/L	1.31 ± 0.32	1.33 ± 0.38	0.063
TG, mmol/L	1.13 (0.81–1.66)	1.10 (0.79–1.58)	0.054
FBG, mmol/L	5.41 ± 0.50	5.39 ± 0.48	0.075
FINS, μIU/mL	9.61 (6.72–13.35)	9.91 (7.33–13.82)	0.124
HOMA-IR	2.33 ± 1.49	2.78 ± 1.60	0.021
hsCRP, mg/L	0.20 (0.03–0.74)	0.65 (0.27–1.33)	0.027
FT3, pmol/L	5.15 ± 0.58	4.94 ± 0.58	0.000
FT4, pmol/L	16.17 ± 1.92	15.76 ± 2.00	0.000
TSH, mIU/L	1.62 (1.20–2.20)	2.11 (1.47–2.98)	0.000
TPOAb, IU/mL	29.10 (0–38.00)	135.75 (46.70–1,248.70)	0.000
TgAb, IU/mL	15.60 (0–22.80)	131.90 (61.30–241.00)	0.000

*Data are means ± SD unless indicated otherwise*.

*TG, FINS, TSH, TPOAb, and TgAb are shown as median and upper and lower quartiles*.

*BMI, body mass index; TC, total cholesterol; LDL-C, low-density lipoprotein cholesterol; HDL-C, high-density lipoprotein cholesterol; TG, triglyceride; FBG, fasting blood glucose; FINS, fasting insulin; HOMA-IR, homeostasis model assessment of insulin resistance; hsCRP, high sensitivity C reactive protein; FT3, free T3; FT4, free T4; TPOAb, antithyroid peroxidase antibodies; TgAb, antithyroglobulin antibodies; AIT, autoimmune thyroiditis*.

### Correlation Between Thyroid Function, Thyroid Antibodies, and Clinical Parameters

Correlation between thyroid function, thyroid antibodies, and clinical parameters is presented in Table 2. FT3 was positively associated with BMI, LDL-C, TG, FBG, FINS, and HOMA-IR and negatively associated with age, TC, and HDL-C (Table 2). FT4 levels were positively associated with LDL-C, TG, and FBG, and negatively associated with age, BMI, HDL-C, and HOMA-IR (Table 2). We also found the positive association between TSH and TC (Table 2). However, these associations lost their significance after the adjustment using Bonferroni correction.

**Table 2:** Correlation between thyroid function, thyroid antibodies, and metabolic parameters.

	FT3		FT4		TSH		TPOAb		TgAb	
	*r*	*P*	*r*	*P*	*r*	*P*	*r*	*P*	*r*	*P*
Age	−0.135	0.000	−0.014	0.000	−0.006	0.660	0.019	0.164	0.004	0.775
BMI	0.182	0.000	−0.050	0.000	0.016	0.105	0.023	0.085	−0.033	0.014
TC	−0.052	0.000	−0.004	0.749	0.029	0.035	0.042	0.002	0.015	0.276
LDL-C	0.032	0.023	0.037	0.008	−0.009	0.524	0.015	0.279	−0.008	0.574
HDL-C	−0.035	0.025	−0.028	0.043	0.016	0.234	−0.008	0.547	0.012	0.316
TG	0.157	0.000	0.082	0.000	0.000	0.987	0.049	0.000	−0.008	0.543
FBG	0.092	0.000	0.052	0.000	−0.023	0.091	0.015	0.271	−0.021	0.127
FINS	0.184	0.000	−0.005	0.328	0.032	0.114	0.063	0.001	0.023	0.266
HOMA-IR	0.188	0.000	−0.052	0.011	0.020	0.332	0.065	0.001	0.027	0.197
hsCRP	0.056	0.657	−0.035	0.779	−0.134	0.286	0.119	0.001	0.063	0.218
FT3	–	–	–	–	–	–	−0.065	0.000	−0.133	0.000
FT4	–	–	–	–	–	–	−0.039	0.004	−0.036	0.008
TSH	–	–	–	–	–	–	0.162	0.000	0.178	0.000

*BMI, body mass index; TC, total cholesterol; LDL-C, low-density lipoprotein cholesterol; HDL-C, high-density lipoprotein cholesterol; TG, triglyceride; FBG, fasting blood glucose; FINS, fasting insulin; HOMA-IR, homeostasis model assessment of insulin resistance; hsCRP, high sensitivity C reactive protein; FT3, free T3; FT4, free T4; TPOAb, antithyroid peroxidase antibodies; TgAb, antithyroglobulin antibodies*.

The TPOAb level was positively associated with TSH, TC, TG, FINS, HOMA-IR, and hsCRP, and negatively associated with FT3 and FT4 (Table 2). The TgAb level was positively associated with TSH and negatively associated with BMI, FT3, and FT4 (Table 2). The levels of TPOAb and TgAb were positively associated with TSH, and negatively associated with FT3 even after Bonferroni correction. Furthermore, after Bonferroni correction, TPOAb still showed a significant association with HOMA-IR and hsCRP (all *P* < 0.05), but the association between TgAb and BMI had disappeared.

### Clinical Characteristics of AIT Groups With Different TPOAb Titers

In the previous studies, the high TPOAb titer was frequently defined when the TPOAb titer was greater than 1,000 IU/mL (17). Therefore, we further divided all AIT patients into two subgroups according to whether their TPOAb titers were greater than 1,000 IU/mL: the low TPOAb AIT group (AIT patients with TPOAb < 1,000 IU/mL) and high TPOAb AIT group (AIT patients with TPOAb ≥ 1,000 IU/mL). The clinical characteristics of the non-obese and euthyroid individuals with different TPOAb titers are summarized in Table 3. There was no significant difference in age, gender, BMI, TC, LDL-C, HDL-C, TG, FBG, and FINS among the control group, the low TPOAb AIT group, and the high TPOAb AIT group. The patients in both the low TPOAb AIT group and the high TPOAb AIT group have decreased FT3, FT4 levels, and increased HOMA-IR, TSH, TPOAb, and TgAb levels than the healthy controls (Table 3). The high TPOAb AIT group has higher hsCRP levels than the control group [0.98 (0.77–1.36) vs. 0.20 (0.03–0.74), *P* < 0.05; Table 3]. The levels of HOMA-IR, hsCRP, FT4, TSH, and TPOAb were significantly increased in the high TPOAb AIT group than the low TPOAb AIT group (Table 3).

**Table 3:** Clinical characteristics of non-obese individuals with different TPOAb titers.

	Control group (*n* = 4,206)	Low TPOAb AIT group (*n* = 1,031)	High TPOAb AIT group (*n* = 371)
Age, years	45.50 ± 12.56	46.21 ± 12.13	45.33 ± 11.95
Gender, F/M	3,098/1,108	751/280	285/86
BMI, kg/m^2^	23.98 ± 2.70	24.03 ± 3.17	23.98 ± 3.23
TC, mmol/L	5.01 ± 0.92	5.02 ± 0.88	4.99 ± 1.00
LDL-C, mmol/L	2.73 ± 0.73	2.74 ± 0.86	2.71 ± 0.68
HDL-C, mmol/L	1.31 ± 0.32	1.34 ± 0.32	1.32 ± 0.35
TG, mmol/L	1.13 (0.81–1.66)	1.11 (0.79–1.57)	1.09 (0.78–1.58)
FBG, mmol/L	5.41 ± 0.50	5.38 ± 0.48	5.40 ± 0.47
FINS, μIU/mL	9.67 (6.74–13.36)	9.74 (7.19–13.85)	10.55 (7.72–13.81)
HOMA-IR	2.33 ± 1.49	2.74 ± 1.60*	2.90 ± 1.58*^§^
hsCRP, mg/L	0.20 (0.03–0.74)	0.41 (0.06–0.65)	0.98 (0.77–1.36)*^§^
FT3, pg/mL	5.15 ± 0.58	4.95 ± 0.61*	4.91 ± 0.50*
FT4, ng/dL	16.17 ± 1.92	15.88 ± 2.00*	15.42 ± 1.95*^§^
TSH, μIU/mL	1.62 (1.20–2.20)	2.00 (1.42–2.82)*	2.46 (1.69–3.32)*^§^
TPOAb, IU/ml	29.10 (0–38.00)	67.55 (35.70–100.03)**	1,300.00 (1,300.00–1,300.00)**^,^^§§^
TgAb, IU/ml	15.60 (0–22.80)	122.30 (55.30–231.30)**	157.45 (76.55–291.65)**

*Data are means ± SD unless indicated otherwise*.

*TG, FINS, TSH, TPOAb, and TgAb are shown as median and upper and lower quartiles*.

*BMI, body mass index; TC, total cholesterol; LDL-C, low-density lipoprotein cholesterol; HDL-C, high-density lipoprotein cholesterol; TG, triglyceride; FBG, fasting blood glucose; FINS, fasting insulin; HOMA-IR, homeostasis model assessment of insulin resistance; hsCRP, high sensitivity C reactive protein; FT3, free T3; FT4, free T4; TPOAb, antithyroid peroxidase antibodies; TgAb, antithyroglobulin antibodies; AIT, autoimmune thyroiditis*.

*The low TPOAb AIT group (AIT patients with TPOAb < 1,000 IU/mL) and high TPOAb AIT group (AIT patients with TPOAb ≥ 1,000 IU/mL)*.

*Significantly different at P < 0.05 vs. the control group.

**Significantly different at P < 0.01 vs. the control group.

*^§^Significantly different at P < 0.05 vs. the low TPOAb AIT group*.

*^§§^Significantly different at P < 0.01 vs. the low TPOAb AIT group*.

### Multivariate Stepwise Regression Analysis

To further evaluate the relationship between TPOAb and HOMA-IR and hsCRP levels, we performed a multivariate regression analysis, including age, gender, BMI, TG, FT3, FT4, TSH, TPOAb, and TgAb. We found that the TPOAb level was an independent influencing factor for HOMA-IR and hsCRP levels (HOMA-IR: β = 0.058, *P* < 0.05; hsCRP: β = 0.108, *P* < 0.05).

## Discussion

This study showed that in a non-obese population with euthyroidism, the patients with AIT had significantly lower FT3 and FT4 levels, and higher TSH, TPOAb, and TgAb levels than the healthy controls. The elevated levels of hsCRP and HOMA-IR were also observed in the AIT patients as compared with the control group. Thyroid function was not associated with metabolic parameters and inflammatory makers, while the TPOAb titer was positively associated with the HOMA-IR and hsCRP levels after adjustment for confounding factors. Multivariate regression analysis demonstrated that the TPOAb level was an independent influencing factor for HOMA-IR and hsCRP levels.

Many epidemiological studies have shown that a certain percentage of AIT patients manifested as euthyroidism (1). Increasing evidence indicated that even AIT patients with euthyroidism still had an increased number of early atherosclerotic lesions (6, 7). This study showed that, although their thyroid function was within the normal range, the non-obese AIT patients had relatively lower FT3 and FT4 levels, and higher TSH than the controls. A recent cohort study from the euthyroid population showed that a low baseline FT4 level was associated with a high risk of the progression of coronary artery calcification over 4 years (18). An *in vitro* study also found that T3 inhibits the calcification and phenotype transformation of vascular smooth muscle cells (19). *In vivo*, recombinant TSH reduces endothelium-dependent vasodilation in patients with differentiated thyroid carcinoma (20). Furthermore, elevated TSH induces endothelial dysfunction in human umbilical vein endothelial cells by inhibiting eNOS expression (21). In euthyroid, patients who underwent coronary angiography, variation of thyroid function within normal range was associated with the severity of coronary atherosclerosis (22). Therefore, relatively lower FT3 and FT4 levels, and higher TSH levels might directly lead to the progression of atherosclerosis.

In this study, non-obese AIT patients with euthyroidism showed higher hsCRP levels than the controls, which suggested a chronic inflammatory state in AIT patients. This result was in line with previous studies. AIT patients had increased hsCRP, IL-6, and IL-1β levels (7, 23, 24). Elevated levels of sICAM-1 and sVCAM-1 were also observed in euthyroid AIT patients compared with the controls (25). Our previous clinical and animal studies showed that the chronic inflammation cause endothelial dysfunction and atherosclerosis (26, 27). Thus, a chronic inflammatory status might be associated with the pathogenesis of atherosclerosis in euthyroid AIT patients.

Autoimmune thyroiditis is characterized by diffuse apoptosis of thyroid follicular cells induced by abnormal cellular and humoral immunity (1, 2). TPOAb, an important antibody for humoral immunity, is mainly produced by lymphocytes in the thyroid (28, 29). AIT patients with increased TPOAb levels have a relatively higher risk for developing hypothyroidism (30). A significant correlation between the TPOAb levels and the number of lymphocytes in the thyroid gland was demonstrated (28). Our study showed that the TPOAb level was positively associated with hsCRP. Previous studies have demonstrated that TPOAb induces cellular cytotoxicity and the complement system and further deteriorated the inflammatory status (2, 28). On the other hand, increased inflammatory factors directly damage thyroid cells, induce more lymphocyte infiltration, and further stimulate the production of TPOAb (2, 29). Carotid intima media thickness (CIMT) is an indicator of early atherosclerosis (31). Several studies have found the positive association between CIMT and thyroid autoantibodies in euthyroid AIT patients (6). Thus, the chronic inflammation may be the link between thyroid autoimmunity and atherosclerosis in AIT patients.

It is well known that hypothyroidism is an important risk factor for metabolic disorders (32). Recently, increased interest has focused on the relationship between thyroid function and metabolic disorders in euthyroid population. This study showed that euthyroid, non-obese AIT patients had higher HOMA-IR levels when compared with the healthy controls. Previous studies have indicated that insulin is critical for a healthy endothelium by inducing the phosphorylation of PI3-K/Akt (33). The activation of the PI3-K/Akt pathway further increased the expression and phosphorylation of eNOS and promoted the production of NO (34). Thus, insulin resistance might contribute to endothelial dysfunction by impairing the PI3-K/Akt pathway and reducing the expression of eNOS and bioavailability of NO. Previous studies have shown that FT4 has an inverse association with HOMA-IR in a euthyroid population with obesity (8, 35). In addition, some studies have found a significant positive correlation between FT3 and hyperinsulinemia (36, 37). An association between TSH and HOMA-IR was also found in some other studies (35). However, most of this evidence came from studies of an obese population or small-scale studies. Obesity not only affects thyroid function but is also a primary risk factor for insulin resistance (12–15). Therefore, obesity might influence the reliability of results. This study showed that FT3 was positively associated with HOMA-IR, while FT4 levels were negatively associated with HOMA-IR. However, these associations lost their significance after adjustment for confounding factors. Consistently, a recent study found an association between thyroid function and metabolic parameters in obese patients with polycystic ovary syndrome (PCOS), while this association disappeared in non-obese PCOS patients (38). These results might suggest that the link between thyroid function and metabolic features is modulated by body weight. Although thyroid function had no relationship with insulin resistance, TPOAb levels were significantly associated with HOMA-IR levels. As mentioned earlier, TPOAb is associated with chronic inflammation. Thus, increased inflammatory status might contribute to increased insulin resistance in non-obese AIT patients with euthyroidism.

## Conclusion

In a non-obese population with euthyroidism, the AIT patients had lower FT3 and FT4 levels, and higher levels of TSH, hsCRP, and HOMA-IR. The TPOAb level is associated with HOMA-IR and hsCRP levels independently of thyroid function. Mild deviation of thyroid function within the normal range, chronic inflammation, and insulin resistance may be the links between AIT and atherosclerosis in the non-obese population. More attention should be focused on AIT patients for cardiovascular disease even if they are euthyroid. And further prospective or intervention researches are needed to evaluate whether levothyroxine treatment will bring benefits in reducing atherosclerosis risk for the AIT patients with euthyroidism, especially in patients with high TPOAb titers.

## Ethics Statement

All enrolled subjects provided written informed consent, and the protocol of this study was approved by the Ethics Committee of the Beijing Chao-Yang Hospital, Capital Medical University.

## Author Contributions

JL and GW conceived and designed the study and wrote the paper; JL, JF, YD, and GW performed the study; JL and JF analyzed the data.

## Conflict of Interest Statement

The authors declare that the research was conducted in the absence of any commercial or financial relationships that could be construed as a potential conflict of interest.
